# Supplementary material for: Arsenophonus symbiosis with louse flies: multiple origins, coevolutionary dynamics, and metabolic significance
Source: mSystems. 2023 Sep 26;8(5):e00706-23. doi: 10.1128/msystems.00706-23 (PMC10654098; doi:10.1128/msystems.00706-23)

Supplementary Figure 1. *Arsenophonus* trees derived from the 10,885 amino acid matrix by Bayesian inference and ML. **A:** Phylobayes (30,000 generations), matrix recoded according to Dayhoff6 scheme. **B:** PHYML under CpREV+R+F model with 100 bootstrap replications. **C:** IQ-TREE under model cpREV+F+I+I+R4 selected by the program (1,000 samples for ultrafast bootstrap). **D:** Q-TREE under mixture model JTT+C60+F+R4 selected by the program (1,000 samples for ultrafast bootstrap). Sequences generated in this study are printed in bold.

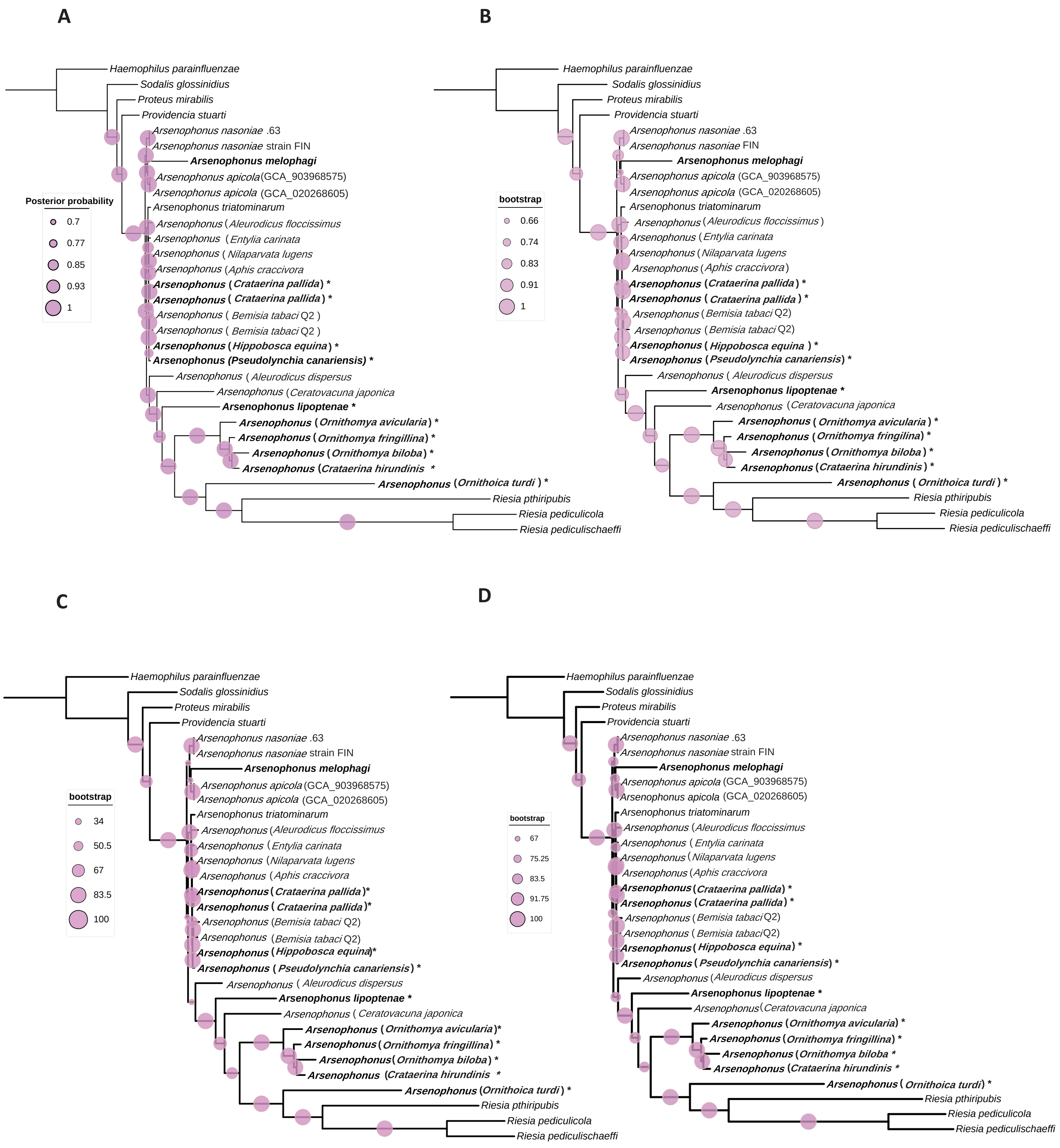

Supplementary Figure 2. PCoA analyses of genome contents calculated using Bray Curtis distances among all protein coding genes (A) and genes with assigned K numbers (B) found across 26 analyzed *Arsenophonus* genomes. Ellipses (not statistical) show obligate (long-branched) strains associated with Hippoboscidae (orange) and lice (pink). The grey-shaded area is explained in the text.

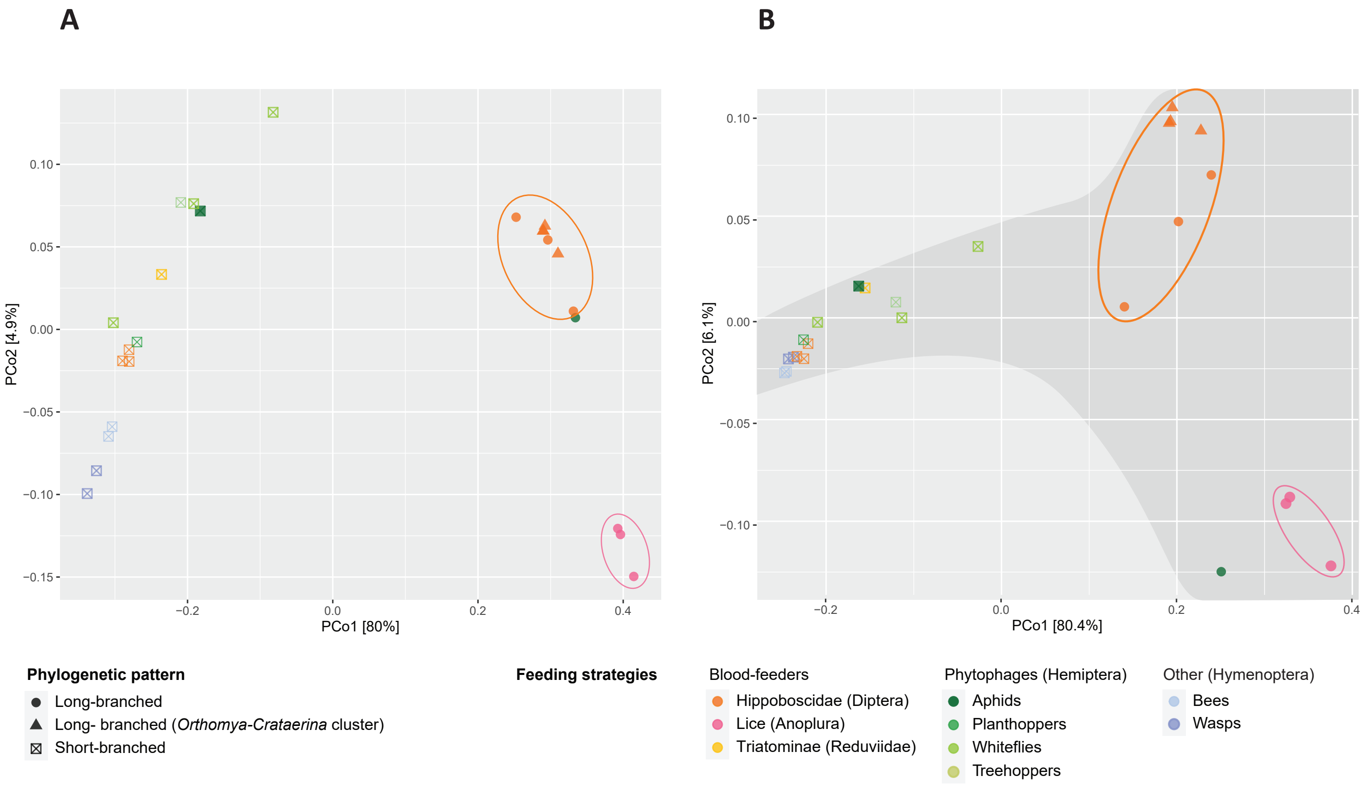

Supplementary Figure 3. Host phylogeny derived from mitochondrial COI gene matrix in IQ-TREE-2 under the GTR+F+I+G4 model with 1,000 ultrafast bootstraps. Sequences generated in this study are printed in bold.

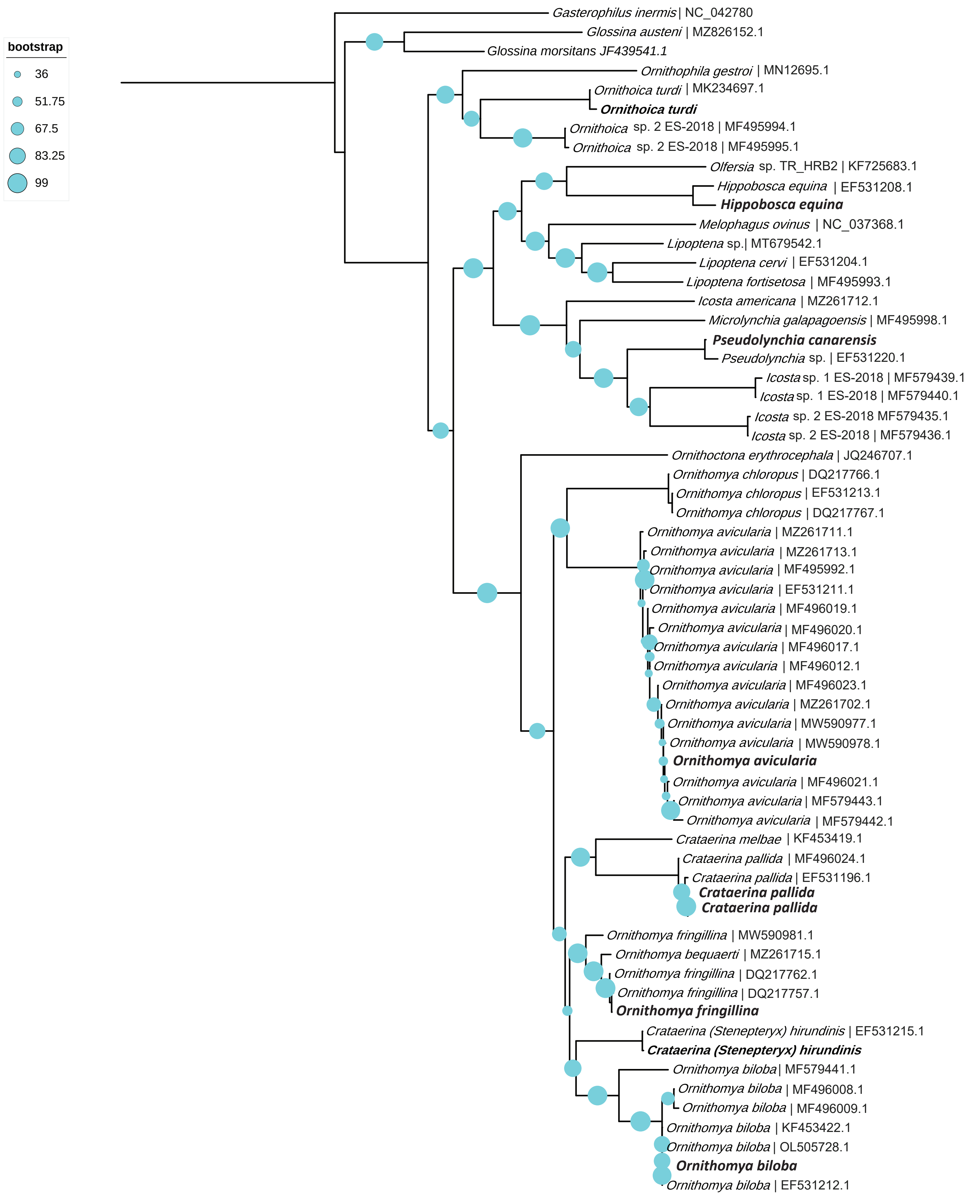

Supplement: Supplemental figures — Fig. S1 to S3. [file msystems.00706-23-s0001.pdf]
